# On the generation of magnetosheath high-speed jets by bow shock ripples

**DOI:** 10.1002/2013JA019172

**Published:** 2013-11-27

**Authors:** H Hietala, F Plaschke

**Affiliations:** 1 Blackett Laboratory, Imperial College LondonLondon, UK; 2 Space Research Institute, Austrian Academy of SciencesGraz, Austria

**Keywords:** magnetosheath, bow shock, forehock

## Abstract

[1]The terrestrial magnetosheath is embedded with coherent high-speed jets of about 1*R*_*E*_ in scale, predominantly during quasi-radial interplanetary magnetic field (IMF). When these high dynamic pressure (*P*_dyn_) jets hit the magnetopause, they cause large indentations and further magnetospheric effects. The source of these jets has remained controversial. One of the proposed mechanisms is based on ripples of the quasi-parallel bow shock. In this paper, we combine for the first time, 4 years of subsolar magnetosheath observations from the Time History of Events and Macroscale Interactions during Substorms mission and corresponding NASA/OMNI solar wind conditions with model calculations of a rippled bow shock. Concentrating on the magnetosheath close to the shock during intervals when the angle between the IMF and the Sun-Earth line was small, we find that (1) 97% of the observed jets can be produced by local ripples of the shock under the observed upstream conditions; (2) the coherent jets form a significant fraction of the high *P*_dyn_ tail of the magnetosheath flow distribution; (3) the magnetosheath *P*_dyn_ distribution matches the flow from a bow shock with ripples that have a dominant amplitude to wavelength ratio of about 9% (∼0.1*R*_E_/1*R*_E_) and are present ∼12*%* of the time at any given location.

## 1. Introduction

[2]The terrestrial magnetosheath is a highly turbulent region between the bow shock and the magnetosphere of the Earth. Within the sheath, observations have revealed striking transient enhancements of plasma flow, denoted as High-Speed Jets (HSJs) in this paper. In various studies they have been characterized by slightly different quantities, such as high dynamic pressure, kinetic energy density, or flux [e.g., *Nemecek et al.*, 1998; *Savin et al.*, 2008; *Hietala et al.*, 2009; *Amata et al.*, 2011; *Archer and Horbury*, 2013; *Plaschke et al.*, 2013].

[3]Two new studies by *Archer and Horbury* [2013] and *Plaschke et al.* [2013] have made significant advances in establishing the statistical properties of HSJs: The HSJ duration in single spacecraft observations varies between a few seconds and a few minutes, a few tens of seconds being typical. Their median spatial scale parallel to the flow is ∼4000km (∼0.6*R*_*E*_). For the size transverse to the flow, multispacecraft observations give estimates of 1200–7000km (0.2–1*R*_*E*_) [*Hietala et al.*, 2009, 2012; *Archer et al.*, 2012]. The dynamic pressure within the jet can be enhanced by a factor of ∼4 compared to the solar wind, and by a factor of ∼15 compared to the ambient magnetosheath. Remarkably, a significant fraction (14.2%) of the HSJs observed in the subsolar region (close to the Sun-Earth line) are supermagnetosonic.

[4]When these jets hit the magnetopause, they can locally distort it [*Shue et al.*, 2009; *Amata et al.*, 2011; *Hietala et al.*, 2012], subsequently setting up magnetopause surface waves (composed of two evanescent magnetosonic waves, one on each side of the boundary) and/or inner-magnetospheric compressional waves [e.g., *Plaschke et al.*, 2009]. Accordingly, in some cases, the effects of the jets have been seen in the magnetosphere all the way to the ground as, e.g., localized magnetic pulsations and ionospheric flow enhancements [*Hietala et al.*, 2012; *Dmitriev and Suvorova*, 2012; *Archer et al.*, 2013].

[5]The only upstream parameter controlling the HSJ occurrence seems to be the interplanetary magnetic field (IMF) cone angle, i.e., the acute angle (∈[0°,90°]) between the IMF and the Sun-Earth line [*Archer and Horbury*, 2013; *Plaschke et al.*, 2013]. Subsolar HSJs occur predominantly during intervals of low cone angle—an IMF geometry that takes place 16% of the time [*Suvorova et al.*, 2010]. They can be found throughout the quasi-parallel magnetosheath when their detection is done by requiring a >100% increase over the ambient magnetosheath dynamic pressure [*Archer and Horbury*, 2013]. When the identification is made by comparison with the solar wind dynamic pressure, the HSJ occurrence probability is largest close to the bow shock and in the sunward half of the sheath [*Plaschke et al.*, 2013]. Based on these statistical results and previous case studies [e.g., *Nemecek et al.*, 1998; *Shue et al.*, 2009; *Hietala et al.*, 2009, 2012], HSJs are thought to be connected to quasi-parallel shock geometry and ion foreshock processes.

[6]Despite this wealth of observational data, the jet generation mechanism has remained unclear and controversial. Based on multispacecraft data from one event, *Hietala et al.* [2009] proposed a mechanism based on bow shock ripples, i.e., local perturbations in the curvature of the shock front that change the angle at which the incoming flow meets the shock. Assuming a high Alfvén Mach number (*M*_A_), a locally inclined shock surface leads to a downstream flow that is compressed but not significantly decelerated. The observational and numerical evidence [e.g., *Schwartz and Burgess*, 1991; *Lucek et al.*, 2008; *Omidi et al.*, 2005; *Blanco-Cano et al.*, 2009] has lead to the perception that large scale foreshock fluctuations steepen as they are convected toward the Earth, dynamically forming and reforming the shock ramp. It thus seems plausible to infer that the quasi-parallel shock is not a smooth surface but inherently rippled.

[7]The recent statistical studies of *Archer and Horbury* [2013] and *Plaschke et al.* [2013] have shown that the majority of the HSJs can not be caused by solar wind discontinuities (or related phenomena), a connection proposed earlier by, e.g., *Lin et al.* [1996], *Archer et al.* [2012], and *Savin et al.* [2012]. However, *Archer and Horbury* [2013] also challenged the ripple-based mechanism: They claimed that the mean deflection of the jet velocity from the nominal background flow—roughly 25° and only few degrees above the ambient turbulence—was inconsistent with the ripple origin.

[8]In this study, we combine for the first time an extensive data set of Time History of Events and Macroscale Interactions during Substorms (THEMIS) and OMNI measurements [*Plaschke et al.*, 2013] with model calculations of bow shock ripples. First, we extend the ripple model, examining how upstream conditions (*M*_A_ in particular) control the jet formation. These results are then juxtaposed with the HSJ observations. Second, we compare the distributions of magnetosheath observations and HSJs near the shock, finding that the jets form a significant fraction of the sheath distribution's high dynamic pressure tail. Third, we perform calculations showing that the downstream flow created by a rippled bow shock is compatible with the observations. Finally, we consider the implications of the results for other plasma environments.

## 2. Models and Data Sets

### 2.1. Modeling Shock Ripples

[9]The two questions we want to address with the modeling are as follows: Is it possible to produce HSJs that are as strong as those observed by locally tilting the shock, when the observed upstream solar wind conditions are taken into account? Is it possible, using model ripples of a certain scale, to produce a flow field that has properties similar to the observations in terms of (a) distribution of the dynamic pressure and (b) distribution of flow deflections? The first question can be answered by generalizing the results of *Hietala et al.* [2009]. The second question requires a three-dimensional model and is statistical in nature. Our approach, described below, is to use simple, point-wise MHD calculations to investigate the statistics of the plasma flow immediately downstream of a rippled shock. To our knowledge, this is the first attempt to quantitatively model the formation of HSJs. In order to simulate the “mesoscale” jets from first principles, one would need both the physics driving shock rippling (i.e., ion reflection) and large enough separation between the kinetic and the global scales. Such sophisticated simulations are not yet available.

**Figure 1:**
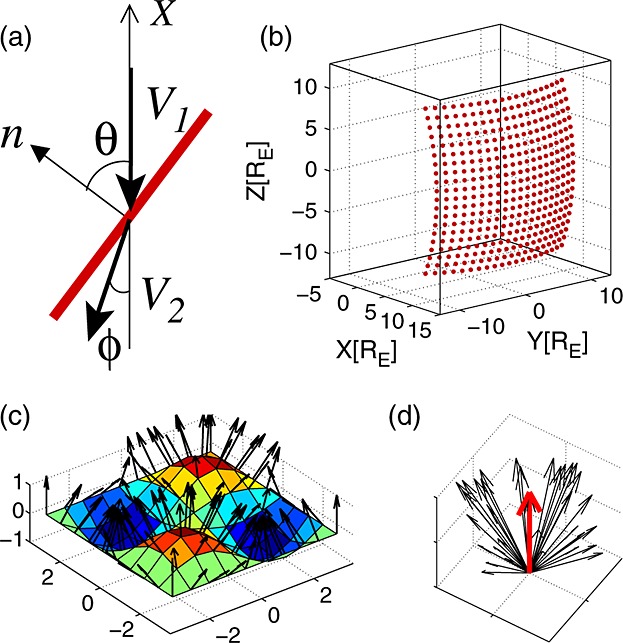
Schematic pictures of the models. (a) The red line depicts the shock with a normal vector n. The black arrows illustrate the plasma velocity upstream (solar wind; lower index 1) and downstream (magnetosheath; lower index 2) of the shock. Magnetic field is aligned with the flow. *X* axis points to the Sun and the shock tilt angle is given by *θ*. The angle between the *X* axis and the downstream velocity is denoted with *φ*. (b) Points on the model bow shock [*Merka et al.*, 2005] (no ripples). (c) Sinusoidal waves (*A*=*k*=1) used to calculate the normal vectors (black arrows) of a rippled surface. (d) Same normal vectors (black arrows) collected around the nominal direction (red arrow).

[10]The basic shock geometry is illustrated in Figure 1a. The (local) tilt angle of the shock is denoted by *θ*. The upstream plasma flow is assumed to be field-aligned (**B**||**V**; exactly radial IMF) so that we operate invariably in the de Hoffmann-Teller frame. Our results are not very sensitive to this assumption. We also assume that the shock is stationary during the formation of the jet. Applying conservation laws to a rippled shock shows that different sections of the surface move at different speeds (in the frame of the upstream plasma) to smooth-out the corrugations [*Prasad*, 2001]. This ripple motion, assumed to take place at a time scale longer than the jet formation, determines the duration of the jet and thus its spatial scale parallel to the flow.

[11]The downstream flow properties can be calculated from the Rankine-Hugoniot jump conditions. The crucial parameter is the shock compression ratio *r*(*θ*,*M*_An_,*β*,*γ*) and its dependence on the shock tilt and the upstream variables: the normal Alfvén Mach number 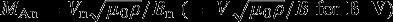, the plasma beta, and the polytropic index *γ* (taken to be 5/3). The index n refers to the vector component normal to the shock. The compression ratio is determined by a cubic equation in *r* that follows from the jump conditions [e.g., *Priest*, 1984]:
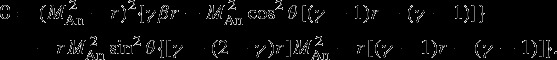
1We calculate *r* from this equation by numerically finding a solution from the fast shock branch using a method similar to *Vainio and Schlickeiser* [1999].

[12]As the shock tilt *θ* is increased, the compression ratio decreases. At a certain large tilt angle that depends on *M*_An_ and *β*, the compression ratio falls below 1 and the solution is no longer physical. In other words, a fast shock can not exist under those conditions. For shock tilts larger than this angle, we set *r*=1, corresponding to no discontinuity.

[13]We focus on high-speed jets that can cause magnetospheric effects, i.e., flows that have an enhanced dynamic pressure in the antisunward direction: 
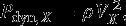
2where *ρ* is the plasma mass density and *V*_*X*_ is the plasma velocity in the Geocentric Solar Ecliptic (GSE) *X* direction. The upstream solar wind is assumed to be purely radial, i.e., to flow along the *X*_GSE_ axis (Figure 1a). In this paper, we concentrate on the behavior of the dynamic pressure ratio *P*_dyn,2,*X*_/*P*_dyn,1,*X*_≡*P*_dyn,MSH,*X*_/*P*_dyn,SW,*X*_ (magnetosheath to solar wind). For the scenario presented in Figure 1a, we can write the dynamic pressure ratio as 
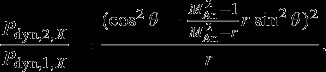
3Note that the ratios of ∼1/*r* and ∼*r* for *θ*∼0° and *θ*∼90° given in *Hietala et al.* [2012] are recovered from equation 3 for *M*_An_≫1. We can see that the ratio does not depend explicitly on the actual values of the upstream velocity, density, and magnetic field, and even the explicit dependence on *M*_An_ is relevant only for *M*_An_≲10. The effect of the upstream parameters comes chiefly through the dependence of *r* on *M*_An_ and *β*.

[14]We are also interested in the deflection angle of ripple-induced flow from the nominal background flow. In the case shown in Figure 1a, the angle *φ* between **V**_2_ and the *X* axis is given by 
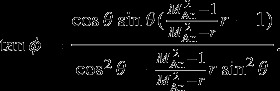
4Thus, the deflection from some nominal downstream flow can be expressed as |*φ*−*φ*_0_|, where *φ*_0_ corresponds to the nominal shock tilt of *θ*_0_. Similarly to the dynamic pressure ratio, we see that the deflection is a function of shock geometry, compression ratio, and Mach number. This is also true in the general three-dimensional case, where the deflection is 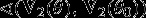.

[15]In order to model the downstream flow statistics of a 3-D bow shock, we first sample 20×20=400 points uniformly from *X*_GSE_=10*R*_*E*_,(*Y*,*Z*)_GSE_∈[−10,10]*R*_*E*_. From there we find the corresponding points on model bow shock [*Merka et al.*, 2005] in the *X*_GSE_ direction (Figure 1b). For model inputs we use generic values of *V*_SW_=400km/s, *n*_SW_=6*cm*^−3^, *B*_SW_=5nT. Note that only the global curvature of the shock (nominal *θ*_0_∈[0°,35.8°]) is relevant for the calculations of this study, and not its position relative to the Earth. We go on to calculate the (background) downstream flow at each point on the shock using the same assumptions as above (**B**||**V**, stationarity).

[16]Next, we need to find the variation of the shock normal vectors around their nominal direction due to rippling. As the observations are an ensemble of multiple single spacecraft passes through the magnetosheath under varying upstream conditions, we are similarly interested in the flow properties caused by the ripples in a statistical sense. For each point on the model bow shock, we sample 100 points uniformly from an arbitrary flat surface *z*=0. We then make sine wave shaped ripples on the surface: *z*=*A* sin(*kx*) sin(*ky*) (Figure 1c). We collect the 100 normal vectors of this rippled surface. This bunch of vectors represents the variability of the shock normal vector at that particular point on the bow shock due to rippling. This is illustrated in Figure 1d by the black vectors around the red vector that indicates the nominal shock normal direction.

[17]The aspect ratio of the waves—the ratio of amplitude to wavelength *A*/*λ*—is a free parameter. Figures 1c and 1d illustrate the case where both *A*=1 and *k*=1, i.e., aspect ratio of *A*/*λ*=*Ak*/2*π*=1/2*π*. Estimates of the spatial size of the HSJs transverse to the flow obtained using multispacecraft observations vary between 0.1–0.5*R*_*E*_ [*Archer et al.*, 2012] and 1–3*R*_*E*_ [*Hietala et al.*, 2009, 2012]. Assuming that the steep section of the ripple of about half a wavelength long produces the jet, we can thus estimate the corresponding amplitude from the model once the aspect ratio is fixed.

[18]We then proceed to calculate the downstream flow properties for each 400×100=40,000 local normal vectors using same assumptions as before (field-aligned flow, stationarity). Each local normal vector provides a “sample” of the flow. Collectively, they describe the flow behind a shock that is rippled in a uniform manner at every point all of the time. These samples are compared with background flow from the smooth bow shock, as well as with the observations.

### 2.2. Observations

[19]We use observations from the five Time History of Events and Macroscale Interactions during Substorms (THEMIS) spacecraft [*Angelopoulos*, 2008] from 2008 to 2011. Namely, we use magnetic field data from the Flux Gate Magnetometer [*Auster et al.*, 2008] and particle data from the Electrostatic Analyzer [*McFadden et al.*, 2008], both originally in spin-period resolution (approximately 3 s cadence). The data are resampled to a common, equidistant time grid of 1 s resolution by linear interpolation. A THEMIS data *sample* is therefore a 1 s interval of interpolated observations. The solar wind conditions for each sample are given by the average of the available NASA OMNI data [*King and Papitashvili*, 2005] from the preceding 5 min. The data sets and the HSJ detection algorithm are described in detail in *Plaschke et al.* [2013]. Here we summarize the main methodology and definitions.

[20]Subsolar magnetosheath data (2736.9h in 6960 individual intervals) are found by selecting times when the THEMIS spacecraft are within 30° of local noon, and between 7 and 18*R*_*E*_ from the Earth. We require that the intervals are at least 2 min long, the ion density exceeds twice the OMNI solar wind ion density, and the energy flux of 1keV ions is larger than the flux of 10keV ions.

**Figure 2:**
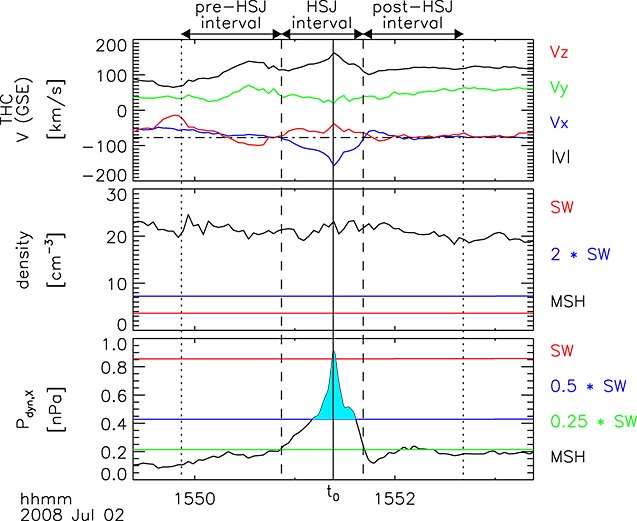
Example of a high-speed jet observed by THEMIS-C on 2 July 2008, illustrating the selection criteria. (top) Ion velocity in GSE coordinates, (middle) ion density, and (bottom) dynamic pressure (using only the *X*_GSE_ component of the velocity). The solid horizontal lines indicate the OMNI solar wind values and their multiples. The light blue shading in Figure 2(bottom) indicates the part of the HSJ interval where *P*_dyn,*X*_ ratio >0.5. Adapted from *Plaschke et al.* [2013].

[21]The main criterion for selecting the HSJs (2859) within the magnetosheath data is that the *P*_dyn,*X*_ ratio shall exceed 0.5, as illustrated in Figure 2. The *jet properties* refer to the time *t*_0_ of the largest *P*_dyn,*X*_ ratio attained by the jet. The interval around this maximum where the *P*_dyn,*X*_ ratio is still larger than 0.25 is called the *HSJ interval*. One minute long intervals before and after the HSJ interval are denoted as *pre-HSJ* and *post-HSJ* data. All three intervals must lie within a magnetosheath data interval, i.e., the jet shall not be too close to a boundary crossing. Additionally, the *X* component of the ion velocity must be negative, and its magnitude shall fall below half of the HSJ velocity within the pre- and post-HSJ intervals.

[22]In this paper, we exclude 1 min intervals from the ends of the magnetosheath intervals (next to boundary crossings). We calculate for all observations their relative distance *r*_rel_ between the model magnetopause (*r*_rel_≡0) [*Shue et al.*, 1998] and the model bow shock (*r*_rel_≡1) [*Merka et al.*, 2005] using the OMNI solar wind conditions as model inputs. To obtain the best correspondence with the model calculations, and in accordance with the findings on occurrence probability [*Archer and Horbury*, 2013; *Plaschke et al.*, 2013], we concentrate on observations close to the bow shock (*r*_rel_>0.5;>0.75) during low IMF cone angles. In the following analysis, we study two subsets of the observations: (a) the sunward half of the sheath, IMF cone angle <45° and (b) the sunward quarter of the sheath, IMF cone angle <30°. The number of samples in each category and subset are given in Table 1. There are 502 HSJs in the first range, and 63 in the second.

**Table 1:** Number of Samples (1s Data Intervals) in the Different Subsets of Data

	MSH^a^(s)	HSJ intervals (s)	HSJs (#)
Whole data set	9,003,852	125,897	2,859
*r*_rel_>0.5, cone angle <45°	278,527	19,092	502
*r*_rel_>0.75, cone angle <30°	30,823	1,970	63

a Excluding 1 min intervals next to boundary crossings.

## 3. Results

### 3.1. HSJs and Upstream Conditions

**Figure 3:**
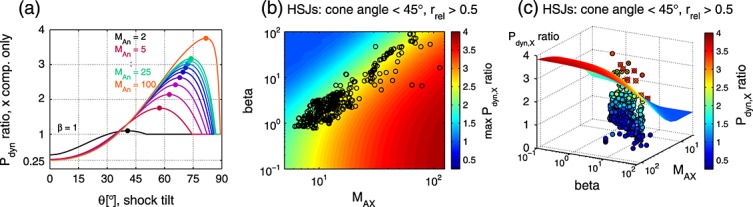
(a) Dynamic pressure ratio as a function of shock tilt *θ* for a range of Alfvén Mach numbers and *β*=1. The maximum *P*_dyn,*X*_ ratios are indicated with dots. (b) Modeled maximum dynamic pressure ratio and HSJs observations. The model calculations are illustrated in color. The black circles indicate the observed upstream conditions for the jets seen at *r*_rel_>0.5 during IMF cone angle <45°. (c) Comparing the observed *P*_dyn,*X*_ ratio with the modeled maximum. The observed ratios are given both by the color and the height of the circle. Those outlying HSJs that are clearly associated with solar wind discontinuities are marked over with a red cross, and the ones with a probable association with a black cross.

[23]Let us first consider the theoretical behavior of the dynamic pressure ratio across the shock as the shock tilt angle *θ* is increased. Figure 3a shows the *P*_dyn,*X*_ ratio (equation 3) as a function of the tilt for *β*=1.0 and a range of Mach numbers: *M*_An_=2 (low Mach number, e.g., interplanetary shocks), *M*_An_= 5–25 (at intervals of 2.5; Earth's bow shock), and *M*_An_=100 (high Mach number, e.g., astrophysical shocks). We find an S-shaped curve, the *P*_dyn,*X*_ ratio first increasing with *θ* and then dropping to 1 when the tilt is too large for the shock to exist. Notably, there is a maximum attainable *P*_dyn,*X*_ ratio that depends on the upstream *M*_An_ and *β*.

[24]Figure 3b shows this maximum *P*_dyn,*X*_ ratio from the model for a range of *M*_An_ and *β* values. The maximum ratio increases with increasing Mach number and decreases with increasing beta. The observed upstream conditions for the 502 HSJs are marked with black circles. We have used 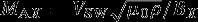 to estimate the shock normal Mach number. The majority of the observed upstream conditions falls roughly on the contour of maximum *P*_dyn,*X*_ ratio of 2.5. Note, however, that this behavior is not a property of the HSJs per se, but a general property of low cone angle solar wind in the data set (not shown).

[25]Are the dynamic pressure ratios of the observed HSJs below the maximum predicted by the model? To investigate this, we have depicted in Figure 3c the observations by circles of color and height appropriate to the measured *P*_dyn,*X*_ ratio, while the modeled maximum *P*_dyn,*X*_ ratio is given by a colored surface. We can see that the vast majority of the observations are below the surface. More precisely, 97% of the observed HSJs have a *P*_dyn,*X*_ ratio below the attainable maximum and are thus producible by ripples.

[26]There are 17 HSJs above the surface (four for *r*_rel_>0.75, cone angle<30°). We checked the solar wind conditions (from OMNI, ACE, and Wind) for these jets. Nine of the outliers are clearly associated with solar wind discontinuities (large red crosses in Figure 3c), and another six are probably related to discontinuities (small black crosses). This leaves two events that are not directly explainable by bow shock ripples nor discontinuities.

[27]We also performed a similar test for 17 events picked randomly below the modeled maximum *P*_dyn,*X*_ ratio surface. Only two of these events had a clear solar wind discontinuity association, and another four had a probable association. The majority of the events (65%) occurred during steady upstream conditions.

### 3.2. Distributions

#### 3.2.1. HSJ-Magnetosheath Comparison

[28]Before comparing the results of the 3-D model with the observations, we want to address a more fundamental question concerning the relationship between the HSJs and the general magnetosheath flow. Do the high-speed jets, though coherent, form the high *P*_dyn,*X*_ ratio tail of the low cone angle magnetosheath? That is, are these two plasma populations in fact the same? What would then be the role of the selection criteria?

**Figure 4:**
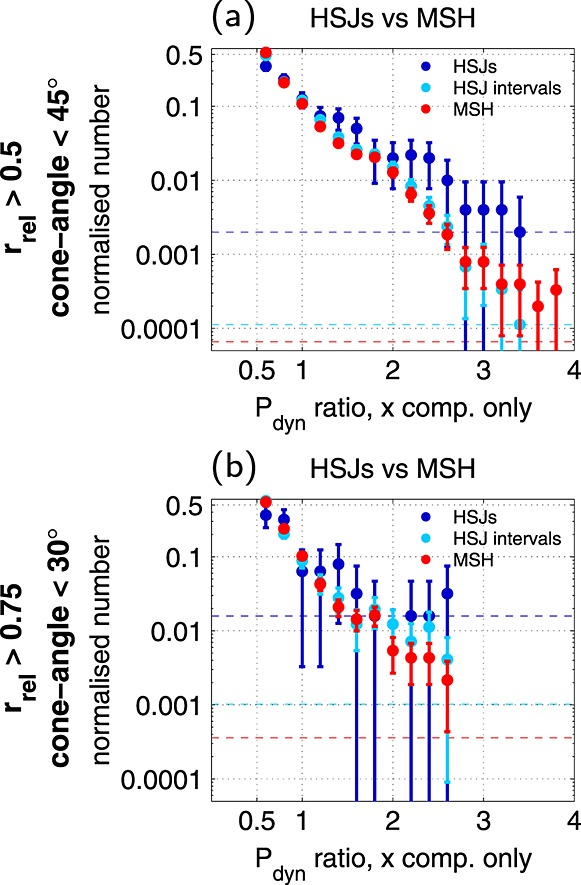
Comparison of observed distributions (a) in the sunward half, cone angle <45° range and (b) in the sunward quarter, cone angle <30° range. The dynamic pressure distribution for samples with *P*_dyn,*X*_ ratio >0.5 from the magnetosheath is displayed in red, and from within the HSJ intervals in light blue. The HSJs (maximum value within an interval) are depicted in dark blue. Note that these distributions include the outlier-HSJs identified in section 3.1. The error bars indicate the 95% binomial proportion confidence interval with the normal approximation and the one-count-levels are given by the horizontal dashed lines.

[29]Figures 4a and 4b compare samples with *P*_dyn,*X*_ ratio >0.5 (the main HSJ criterion) taken from the magnetosheath data set (red) and from within the HSJ intervals (light blue; see also the shading in Figure 2). This high *P*_dyn,*X*_ ratio tail constitutes 5.4% of the magnetosheath samples in the sunward half, cone angle <45° case, and 9.0% in the sunward quarter, cone angle <30° case. The distribution of the *P*_dyn,*X*_ ratio >0.5 samples that belong to HSJ intervals is practically identical to the magnetosheath samples in the first range. Closer to the bow shock, there is more scatter, but this may be due to lower amount of samples. The two distributions are still very similar.

[30]The role of the other selection criteria (jet-like *V*_*X*_ profile, well separated from boundary crossings) is demonstrated by the fact that the fraction of the *P*_dyn,*X*_ ratio >0.5 sheath data that belongs to the HSJ intervals is not 100%. For *r*_rel_>0.5 and cone angle <45°, it increases with *P*_dyn,*X*_ ratio from ∼55% to ∼75%. For *r*_rel_>0.75 and cone angle <30°, the fraction increases from ∼35% to ∼80%. Thus, coherent jet-like enhancements form a significant fraction of the tail. Furthermore, it is not surprising that the typical fraction is somewhat larger for the first subset since the HSJ identification is made by comparison with the solar wind dynamic pressure. It is expected that as the nominal plasma slows down within the sheath, a larger and larger fraction of high *P*_dyn,*X*_ ratio observations begins to correspond to jets that have penetrated into that region. However, it is interesting to note that the strongest dynamic pressure enhancements seen in the *r*_rel_>0.5, cone angle <45° range do not belong to HSJ intervals (Figure 4a).

[31]The dark blue distributions of the HSJs correspond to the maximum values within each HSJ interval. This act of selection introduces some clear differences to the distributions, as there seems to be an increase in values near 1.5 and 2.3 compared to the sheath and the HSJ intervals. Note that we have not excluded the outliers identified in section 3.1. Hence, the last six(three) bins above *P*_dyn,*X*_ ratio =2.3(2.0) in Figure 4a(4b) are heavily affected by them.

[32]We conclude that during intervals of low IMF cone angle, the HSJs do form a significant fraction (35–80%) of the magnetosheath high *P*_dyn,*X*_ ratio tail. In other words, the magnetosheath variability during low cone angles is such that the plasma is spatially and temporally concentrated into jets, as opposed to random fluctuations. However, differences in distributions can arise depending on the value (maximum, mean, etc.) chosen to represent a jet interval.

#### 3.2.2. Magnetosheath-Model Comparison

[33]We now proceed to compare the observations with the flow statistics obtained with the 3-D model, as described in section 2.1. Since we found that the observations roughly follow the max *P*_dyn,*X*_ ratio=2.5 contour in Figure 3b, it suffices to use a representative combination of the input upstream *M*_An_ and *β* values that fulfills this condition. In this study, we have chosen *M*_An_=15 and *β*=2.5, that are near the median values of the magnetosheath data set (*M*_A*X*_=12.4 and *β*=2.5 for *r*_rel_>0.5, cone angle <45°).

[34]Based on the results of the previous subsection, it is also most reasonable to compare the model flow with the general magnetosheath distribution. The main constraint comes from the fact that while the Rankine-Hugoniot conditions state that the *P*_dyn,*X*_ ratio is larger than 0.25 immediately downstream of the shock, deeper in the sheath, the plasma undergoes further deceleration and the flow is deflected even more. Consequently, the *P*_dyn,*X*_ ratio >0.25 magnetosheath observations constitute only 24% of the samples in the sunward half, cone angle <45° range, and 32% in the sunward quarter, cone angle <30° range.

**Figure 5:**
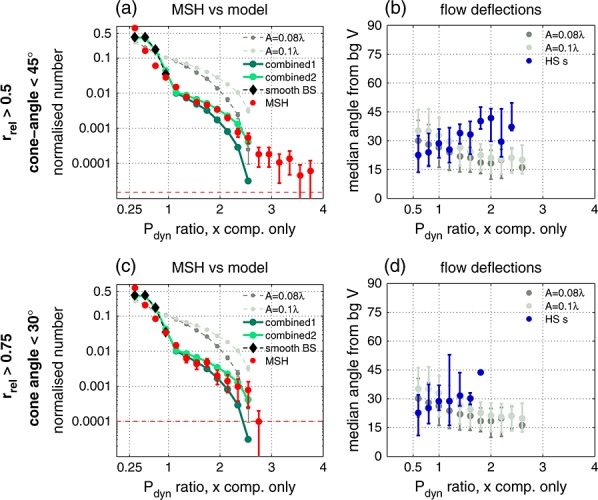
Comparison of observed and modeled distributions (top) in the sunward half, cone angle <45° range, and (bottom) in the sunward quarter, cone angle <30° range. (a,c) Dynamic pressure distributions for samples with *P*_dyn,*X*_ ratio >0.25 from the magnetosheath (red) and different 3-D models. Distribution from a smooth bow shock is displayed by black diamonds, while results for uniformly rippled shock with aspect ratios *A*/*λ*=8*%* and 10% are in dark and light gray. Combinations with a weighting of 7:1 between the smooth and rippled shock distributions are shown in green (*A*/*λ*=8*%*, dark; *A*/*λ*=10*%*, light). The error bars indicate the 95% binomial proportion confidence interval with the normal approximation, and the one-count-levels are given by the horizontal dashed lines. (b,c) Median deflections from the background velocity for two models (*A*/*λ*=8*%* and 10%; dark and light gray) and the HSJs. See text for details. The outlier HSJs identified in section 3.1 have been excluded from these two distributions. The error bars indicate the upper and lower quartiles.

[35]Figures 5a and 5c show the magnetosheath distributions (red) together with the model results. The slope of the sheath distribution clearly changes near *P*_dyn,*X*_ ratio∼1. Close to the bow shock (Figure 5c) the distribution falls sharply near 2.5, while deeper in the sheath (Figure 5a) there are some observations above it. Partly, this is due to the statistics (note the one-count-levels). Some of these *P*_dyn,*X*_ ratio >2.5 samples belong to the solar wind discontinuity related HSJs (Figure 3c), and some may be caused by additional processing of the plasma, such as focusing of the flow.

[36]Model calculations for a smooth bow shock (black diamonds) produce a core that does not exceed *P*_dyn,*X*_ ratio ∼1. Introducing ripples with an increasing aspect ratio (*A*/*λ*=8*%* in dark gray, 10% in light gray) leads to wider distributions extending eventually up to the maximum ratio 2.5. Given that we found that the fraction of data belonging to the HSJ intervals increases with increasing *P*_dyn,*X*_ ratio (section 3.2.1), this brings further evidence in favor of the ripple origin of the jets.

[37]The model distributions (gray) describe a shock where every point is equally rippled all of the time. We can represent intermittent rippling by varying the weighting between distributions from a smooth shock and a rippled shock. Using a weighting of 7:1 for aspect ratios of 8 and 10% (“combined1,” dark green; “combined2,” light green), we find two shapes that are compatible with the observations. Note that these two combined models fit well both of the subsets. The low *P*_dyn,*X*_ ratios match better closer to the bow shock (*r*_rel_>0.75), in agreement with the model assumptions.

[38]To conclude, the dynamic pressure ratio distribution produced by ripples that are present ∼12% of the time matches quite well the observed magnetosheath (and HSJ) distribution. We find that the primary aspect ratio of the ripples is ∼9%. If scaled with the observational estimate of jet transverse size of about 0.5–1*R*_*E*_, this corresponds to ripples with an amplitude of 500–1000km.

#### 3.2.3. Deflections

[39]Finally, we compare the behavior of the flow deflections. For each point on the model bow shock, we calculate the angles between the nominal flow direction (smooth bow shock) and the velocity vectors resulting from the ripples (Figure 1c). These are then binned according to the *P*_dyn,*X*_ ratio. Estimating the nominal flow direction for individual HSJs from observations is difficult; here we use the average of the pre- and post-HSJ flow directions. We also exclude those HSJs that were identified as outliers in section 3.1.

[40]The act of adding bow shock ripples introduces deflections to the magnetosheath flow for all *P*_dyn,*X*_ ratios, as shown in Figures 5b and 5d. Increasing the ripple amplitude increases the median deflections so that the values for aspect ratios of 8–10% are between ∼15° and ∼35°. The highest *P*_dyn,*X*_ ratios have lower median deflections than the lower *P*_dyn,*X*_ ratios.

[41]The observed median deflections for HSJs are between ∼20° and ∼45°. The values for low *P*_dyn,*X*_ ratio HSJs are similar to *Archer and Horbury* [2013]; their superposed epoch analysis was dominated by these small dynamic pressure enhancements that are more numerous. Close to the bow shock in the *r*_rel_>0.75 range, the deflection distribution is quite flat (given the spread in the upper and lower quartiles), and the model results are quantitatively close to the observations (Figure 5d). However, the more we include observations taken deeper in the sheath, the larger the deflections become for the higher *P*_dyn,*X*_ ratios. This results in the clear increasing trend in Figure 5b. Close to the magnetopause (*r*_rel_<0.25), the median deflections for high *P*_dyn,*X*_ ratio (2.5–4) jets are in fact as large as 50–70°.

[42]The emergence of a trend showing increased deflections with depth in the sheath is consistent with strong HSJs keeping their direction (and eventually hitting the magnetopause) while the nominal magnetosheath plasma flows around the magnetosphere. Unfortunately, we do not have enough measurements at *r*_rel_≲1 (i.e., right behind the shock) to resolve whether or not the deflection distribution is slightly decreasing immediately downstream of the shock similarly to the model. Nevertheless, it is clear that the ripple mechanism does not prescribe strong jets to have larger deflections than the overall directional variability, in contrast to the claim made by *Archer and Horbury* [2013].

## 4. Discussion

[43]The analysis in section 3.2.1 revealed that a significant fraction or even majority of the magnetosheath high dynamic pressure ratio tail corresponds to coherent jets. Above all, we find that the differences between the HSJ and the magnetosheath distributions are consequences of the selection criteria, in particular, that of choosing the maximum *P*_dyn,*X*_ ratio within each HSJ interval to represent the jet.

[44]Our 3-D modeling of a rippled bow shock provided a flow distribution that is compatible with the magnetosheath observations. Let us briefly summarize the factors defining the shape of this distribution. The distribution can extend up to a maximum *P*_dyn,*X*_ ratio that is imposed by the upstream conditions. Based on the results illustrated in Figure 3b, this is typically around 2.5, and we modeled it using *M*_An_=15 and *β*=2.5. The aspect ratio of the ripples then determines whether or not this upper limit is reached, as well as the slope of the distribution. The relative amount of the high dynamic pressure observations determines the weighting between the core produced by the smooth shock and the tail produced by the rippled shock.

[45]Considering first the ripple aspect ratio, we note that in nature there is probably a distribution of ripple aspect ratios (or amplitudes). The modes of *A*/*λ*=0 and 9% found here correspond to the most prominent components of this distribution. For the 9% aspect ratio, the maximum angular variation of the shock normal direction is ∼32°. This degree of variation as well as the scaled amplitude of 500–1000km can be directly compared with multispacecraft observations and simulations in the future. Better understanding on the details of how foreshock structures form the shock and perturb it is needed. At the moment, we can note that the spatial scales found in this study for the ripples are quite similar to the scales over which Short Large Amplitude Magnetic Structures (SLAMS) are observed to be coherent, and the thickness of the shock transition layer [*Lucek et al.*, 2008].

[46]Second, the low cone angle magnetosheath *P*_dyn,*X*_ distribution seems to consist of two main populations: a core from smooth shock and a tail of mostly coherent jets from rippled shock. There is a number of ways to interpret the 7:1 weighting between them. All of the subsolar bow shock surface could be rippling simultaneously at quasi-regular intervals. Such activity might be related to foreshock ULF waves “breaking on the shore” (i.e., the shock), since they have been observed to have a transverse correlation scale that is of the same order as the size of the bow shock [*Archer et al.*, 2005]. Similarly, a particular region constituting 12% of the subsolar bow shock surface may be continuously rippled. This could be, e.g., the region near the foreshock edge. Naturally, it cannot be excluded that the behavior is due to a particular solar wind feature that has not been considered in this or the previous studies that occurs about 12% of the time when the cone angle is low.

[47]The interpretation we find most plausible is that the ripples are spatially random, i.e., appearing and disappearing as the foreshock waves and structures are convected to the shock. Thus, the conclusion from the distributions would be that any given point on the subsolar shock surface is rippled about 12% of the time, on average, when the IMF cone angle is low. Accordingly, a single spacecraft in the magnetosheath should record an inter-HSJ duration that is about seven times longer, on average, than the HSJ intervals. From a time series point of view, this is rather consistent with the results of *Plaschke et al.* [2013] stating that while the median duration of a HSJ is 29s, the median length of time between two HSJ within a common sheath interval is 140s. Note, however, that this is not a completely independent confirmation as both estimates are derived from the same data set. It also agrees with the occurrence rate of ∼10% of the time close to the quasi-parallel shock obtained by *Archer and Horbury* [2013], again from THEMIS observations.

[48]The results displayed in Figures 3a and 3b concerning the maximum attainable *P*_dyn,*X*_ ratio are readily applicable to many space plasma environments. Typical interplanetary shocks at 1AU, for instance, are not expected to have strong HSJs since they tend to have Mach numbers of less than 3 [*Echer et al.*, 2003]. Saturn's bow shock, on the other hand, can have a very high Mach number [*Masters et al.*, 2011]. In the event recently described by *Masters et al.* [2013], Cassini spacecraft crossed the shock during *M*_A_∼100, electron *β*∼10, and field-aligned flow. We would expect strong ripple induced jets under such conditions. Still, it is unclear whether their transverse size would be sufficient to cause significant effects on the vast magnetosphere of the gas giant.

[49]At the edge of the solar system, the heliospheric termination shock is generally quasi-perpendicular, but may exhibit rippling [*Burlaga et al.*, 2008]. Interestingly, MHD simulations by *Opher et al.* [2003, 2004] of the interaction between the shock and the heliospheric current sheet have revealed formation of a high-speed jet. The authors concentrated on the behavior of this jet deeper in the heliosheath and possible acceleration by the de Laval nozzle effect. However, it is evident from their figures that the origin of the jet and its converging streamlines is a ripple on the shock, probably caused by the heliospheric current sheet.

[50]It would also be interesting to study ripples on the possible quasi-parallel, slow bow shock of the heliosphere [*Zieger et al.*, 2013]. For that purpose the model calculations presented here should be modified so that the solution for the compression ratio (equation 1) would be chosen from the slow shock branch instead of the fast shock branch. Thus, the effects of the ripples might be different compared to fast bow shocks.

## 5. Conclusions

[51]In this paper, we have presented the first quantitative study on the connection between magnetosheath high-speed jets and quasi-parallel bow shock ripples. During low IMF cone angle conditions, the magnetosheath flow distribution near the bow shock is compatible with the distribution arising from shock ripples of *A*/*λ*∼9*%* (∼0.1*R*_*E*_/1*R*_*E*_), present ∼12*%* of the time at a given location. These ripples increase flow deflections for both small and large dynamic pressure ratios, and the median values for HSJs agree quantitatively with the model results very close to the shock. Quite remarkably, we find that the coherent HSJs largely form the high dynamic pressure ratio tail of the sheath distribution. It seems that they may occur whenever the cone angle is low, and 97% of the HSJs observed near the bow shock during low cone angles can indeed be produced by ripples under the observed upstream conditions. Their maximum attainable strength has a strong dependence on the solar wind Mach number and plasma beta.

[52]Despite being part of the overall magnetosheath flow distribution, the ripple origin makes the jets spatially coherent structures, as opposed to random fluctuations. Thus, their effects when impinging on the magnetopause are distinct, as seen in previous studies. This coherent nature warrants their study as a distinct phenomenon. A relatively simple model of the bow shock ripples gives a rather good agreement with the observations, and encourages future work using full simulations.
